# Luteinizing Hormone-Releasing Hormone (LHRH)-Conjugated Cancer Drug Delivery from Magnetite Nanoparticle-Modified Microporous Poly-Di-Methyl-Siloxane (PDMS) Systems for the Targeted Treatment of Triple Negative Breast Cancer Cells

**DOI:** 10.3390/jfb15080209

**Published:** 2024-07-28

**Authors:** Stanley C. Eluu, John D. Obayemi, Danyuo Yiporo, Ali A. Salifu, Augustine O. Oko, Killian Onwudiwe, Toyin Aina, Josephine C. Oparah, Chukwudi C. Ezeala, Precious O. Etinosa, Sarah A. Osafo, Malachy C. Ugwu, Charles O. Esimone, Winston O. Soboyejo

**Affiliations:** 1 Department of Pharmaceutical Microbiology and Biotechnology, Nnamdi Azikiwe University, Ifite Awka 420110, Nigeria; 2 Department of Biotechnology, Ebonyi State University, Abakaliki 480101, Nigeria; 3 Department of Mechanical and Material Science Engineering, Higgins Lab, Worcester Polytechnic Institute (WPI), 100 Institute Road, Worcester, MA 01609, USA; 4 Department of Biomedical Engineering, Worcester Polytechnic Institute, Gateway Park Life Sciences and Bioengineering Centre, 60 Prescott Street, Worcester, MA 01609, USA; 5 Department of Mechanical Engineering, Ashesi University, Berekuso PMB CT3, Ghana; 6 Department of Mechanical Engineering, Academic City University College, Haatso-Accra P.O. Box AD 421, Ghana; 7 Department of Engineering, Morrissey College of Arts and Science, Boston College, Chestnut Hill, MA 02467, USA; 8 Department of Biology and Biotechnology, David Umahi Federal University of Health Sciences, Uburu 480101, Nigeria; 9 Aerospace and Mechanical Engineering, University of Notre Dame, Notre Dame, IN 46556, USA; 10 Department of Biomedical Engineering, Collage of Engineering, Afe Babalola University, KM 8.5 Afe Babalola Way, Ado-Ekiti 360001, Nigeria; 11 Department of Material Science, African University of Science and Technology, Km 10 Airport Road, Abuja 900107, Nigeria; 12 Department of Biotechnology, Worcester State University, Worcester, MA 01602, USA; 13 Department of Material Science and Engineering, University of Ghana, Legon, Accra P.O. Box LG 1181, Ghana; 14 Biomaterial Science Department, Dental School, College of Health Sciences, University of Ghana, Korle bu, Accra P.O. Box KB 52, Ghana; 15 Department of Engineering, SUNY Polytechnic Institute,100 Seymour Rd, Utica, NY 13502, USA

**Keywords:** conjugated drugs, sustained drug release, cell viability, triple-negative breast cancer, magnetite, poly-di-methyl-siloxane (PDMS), targeted and localized treatment

## Abstract

This study presents LHRH conjugated drug delivery via a magnetite nanoparticle-modified microporous Poly-Di-Methyl-Siloxane (PDMS) system for the targeted suppression of triple-negative breast cancer cells. First, the MNP-modified PDMS devices are fabricated before loading with targeted and untargeted cancer drugs. The release kinetics from the devices are then studied before fitting the results to the Korsmeyer–Peppas model. Cell viability and cytotoxicity assessments are then presented using results from the Alamar blue assay. Apoptosis induction is then elucidated using flow cytometry. The in vitro drug release studies demonstrated a sustained and controlled release of unconjugated drugs (Prodigiosin and paclitaxel) and conjugated drugs [LHRH conjugated paclitaxel (PTX+LHRH) and LHRH-conjugated prodigiosin (PG+LHRH)] from the magnetite nanoparticle modified microporous PDMS devices for 30 days at 37 °C, 41 °C, and 44 °C. At 24, 48, 72, and 96 h, the groups loaded with conjugated drugs (PG+LHRH and PTX+LHRH) had a significantly higher (*p* < 0.05) percentage cell growth inhibition than the groups loaded with unconjugated drugs (PG and PTX). Additionally, throughout the study, the MNP+PDMS (without drug) group exhibited a steady rise in the percentage of cell growth inhibition. The flow cytometry results revealed a high incidence of early and late-stage apoptosis. The implications of the results are discussed for the development of biomedical devices for the localized and targeted release of cancer drugs that can prevent cancer recurrence following tumor resection.

## 1. Introduction

Breast cancer, with over 2.3 million annual cases globally, ranks as the most prevalent cancer in adults and affects 95% of nations [1]. It represents a significant global health challenge, with various subtypes, each having unique biological traits that cause different responses to treatments and clinical outcomes [2]. Progesterone receptor (PR), estrogen and progesterone receptor (ER and PR), and human epidermal growth factor receptor 2 (HER2) expression levels are used to guide conventional therapy methods for certain individuals [3]. Triple-negative breast cancer (TNBC) differs from other subtypes because of its aggressive behavior and few available treatment options. 

TNBC is characterized by the absence of HER2, PR, and ER expression, making it unresponsive to specific therapies that target these receptors, including hormone-based therapy and HER2-targeted drugs [4]. Hence, when these drugs are administered, they are distributed throughout the body and systemic circulation, and only a small percentage of the medications reach the tumor site. 

Hence, individuals diagnosed with TNBC have reduced survival rates compared to other types of breast cancer, with a 40% mortality rate within the initial five years after diagnosis [5]. TNBC is quite invasive, and 46% of patients will experience distant metastases [6]. Solid primary tumors that are well-defined, accessible, and located in nonvital tissue regions can be successfully removed through conventional surgery, but residual tumor tissues are typically also left behind [7]. Furthermore, because surgical removal entails significant risks and consequences, this treatment is not suitable for treating small, poorly defined metastatic tumors located in critical areas [8]. 

However, chemotherapy remains the primary systemic treatment, even though it has poor efficacy and often exacts indiscriminate effects on both cancerous and healthy cells due to its non-selective nature. The enormous damage that chemotherapy medications cause to healthy tissues and organs is a primary factor contributing to the high mortality rate in cancer patients [9]. Considering TNBC’s resistance to standard treatment and the urgent need to improve patient outcomes, the unrelenting search for an effective treatment for the disease continues to be a top priority in the field of cancer research. Hence, there is a need for new approaches to tackle the specific issues presented by this aggressive subtype of breast cancer.

The development of targeted and controlled drug delivery systems has shown promise for improving the specificity and effectiveness of cancer treatment while reducing side effects on healthy tissues [10,11]. Research has shown that the drug delivery system’s ability to simultaneously load, distribute, and release chemotherapeutic drugs, along with its precise targeting capabilities, allows for the highly effective and targeted treatment of TNBC by effectively inhibiting and destroying tumor cells [12]. One significant advancement in this area is the incorporation of magnetic nanoparticles, particularly magnetite nanoparticles, into drug delivery systems. These nanoparticles have distinctive qualities, like programmable magnetic responses and biocompatibility, that can be used to direct and improve drug delivery strategies [13,14,15].

Furthermore, microporous Poly-Di-Methyl-Siloxane (PDMS) systems have gained prominence in biomedical engineering for their versatility, biocompatibility, excellent oxidation resistance, minimal toxicity, exceptional flexibility, ease of production, cost-effectiveness, permeability to gases, and reduced surface tension [15,16,17,18]. These systems can act as optimal vehicles for drug delivery, enabling sustained and controlled drug release. A suitable drug delivery device can be created by adding magnetite nanoparticles to the PDMS matrix, enabling controlled drug delivery. 

In this paper, we report the fabrication of magnetite nanoparticle-modified microporous PDMS for the delivery of conjugated and unconjugated drugs for the targeted treatment of TNBC. Biocompatible targeting molecules, such as aptamers, antibodies, and peptides that are specific to the antigens or receptors at the tumor site, are conjugated to the surface of drugs in active targeting [10,19]. To enhance the precision of drug delivery and target tumor cells effectively, we first conjugated these drugs with luteinizing hormone-releasing hormone (LHRH). LHRH has been recognized for its role in the negative autocrine/paracrine regulatory mechanism of cell proliferation, with expression in various malignant tumors, including endometrial, ovarian, and triple-negative breast cancers (TNBC) [20,21,22]. This targeting moiety exhibits high specificity for receptors present at the tumor site, ensuring a targeted and efficient drug delivery mechanism. By combining these strategies, our study aims to provide a comprehensive and precise approach to breast cancer treatment, offering new possibilities to bring us closer to more effective treatments for this aggressive subtype of breast cancer.

## 2. Materials and Methods

### 2.1. Materials

The process of synthesizing prodigiosin involved sourcing various materials and reagents from different suppliers. Fetal bovine serum (FBS), penicillin-streptomycin, and Leibovitz’s media were obtained from the American Type Culture Collection (ATCC, Manassas, VA, USA). Thermo Fisher Scientific (Waltham, MA, USA) supplied paclitaxel, N-hydroxysuccinimide (NHS), 1-ethyl-3-(3-dimethylaminopropyl) carbodiimide hydrochloride (EDC HCl), Alamar Blue Assay kits, Dubecco Phosphate Buffer (DPBS), 12-well plates, and opaque 96-well plates. Additionally, we acquired the Amicon Pro Purification System and three kDa Amicon Ultra-4 Centrifugal Filter Units from Millipore Sigma (Burlington, MA, USA). A cross-linker and the Sylgard^®^ 184 silicone elastomer kit was purchased from Dow Corning Corporation in Midland, TX, USA, while magnetite (iron oxide; Fe3O4) nanopowder (high purity, 99.5%, 15–20 nm size range) was purchased from US Research Nanomaterials (Houston, TX, USA). The synthesized prodigiosin designated for conjugation with LHRH displayed a purity of 92.5%.

### 2.2. Experimental Methods

#### 2.2.1. Preparation of Conjugated Drugs 

Preparation of conjugated drugs was carried out as reported in our previous work [21]. Prodigiosin and paclitaxel were each conjugated with luteinizing hormone-releasing hormone (LHRH). Prodigiosin (PG) is also referred to as 4-methoxy-5-[(Z)-(5-methyl-4-pentyl-2H-pyrrol-2-ylidene) methyl]. One of the methoxy substituents in the 4-methoxy-2,20-bipyrrolyl ring of -1H,1′H-2,2′-bipyrrole is C-6. PG’s methoxy group, characterized by a high electron density and a propensity to attack the nucleophilic core of the carbonyl group in the NHS linker, was meant to interact with the hydrophilic linker NHS by acting as reactive sites. The proton from the N–H group was electrostatically cleaved due to attacks on PG linkages in the presence of EDC, which increased electron density. This made it easier for LHRH to bind, making it difficult to break amide bonds. NHS ester crosslinks were formed in the presence of LHRH molecules, binding to the α- and ε-amines in the N-terminals and the lysine side chains.

For paclitaxel, the drug coupling specifically focused on the inherent lysine ε-amines groups within the LHRH peptide (Equations (1) and (2)).

(1) $$OH-2^{\prime }-PTX+Succinic\;Anhydride\;\to PTX-2^{\prime }-O_{2}PTX{\;O}_{2}\;OCCH_{2}\;CH_{2}\;CO_{2}\;H\;\left(PTXSCT\right)\;$$

(2) $${LHRH-NH}_{2}+PTXSCT\;\to \frac{NHS/EEDG}{DMF}\to LHRH-NH-PTX\;(PTX-LHRH)$$

The targeted components were joined to PTX using the 2-hydroxyl group on one of its side chains through the use of heterobifunctional linkers. These linkers firmly join the drug segment and the LHRH peptide so that the ligands can be accurately affixed to the target receptors on tumors or cancer cells.

#### 2.2.2. Fabrication of Drug-Loaded MNP-Modified Microporous PDMS System and Nonporous MNP-Modified PDMS Structure

##### Fabrication of Drug Loaded MNP-Modified Microporous PDMS System

Poly-n-isopropyl-acrylamide (PNIPA) hydrogels were produced via additional polymerization, as described in prior work [23]. Then, a silicone elastomer base (Sylgard 184) was combined with a silicone elastomer curing agent in a 10:1 volume ratio to create PDMS substrates. The mixture was thoroughly stirred to ensure uniformity. This mixture was degassed in a vacuum oven (Isotemp vacuum oven, model 280A, Fisher Scientific, Waltham, MA, USA) at −20 inHg for an hour to reduce volume defects. The mixtures were combined with 5 wt.% magnetite nanoparticles (5 wt.%MNP) and degassed. Drops of the resulting mixtures were gently poured into a rectangular plastic mold to fill the bottom. 

Sugar cubes were partially submerged in the PDMS_MNP blends within the mold. To make delamination easier during remolding, aluminum foil was used to separate the sugar cubes. The polymer mixtures were cast over the sugar cubes until saturation, allowing the MNP+PDMS to permeate into their porous architecture. Excess air bubbles induced during the casting stage were removed by degassing the samples for 15 min. Subsequently, the samples were cured at 60 °C for 4 h and left to cool to room temperature (25 °C) before the sugar cube structures were gently removed using surgical blades. The samples were placed in fresh double-deionized water (DDW) with a volume of 1000 mL and leached for five days, replacing the DDW every 6 h until all the sugar was removed entirely from the polymer matrix. The leached MNP+PDMS formed foams for further characterization and complete device encapsulation.

The drug carrier (PNIPA hydrogels) was integrated into the inner core of the MNP+PDMS structures and then precisely cut to measurements of 0.106 mm in thickness, 1.15 mm in width, and 1.31 mm in length. The porous encapsulated devices (MNP+PDMS-PNIPA-based devices) were sterilized as appropriate. Unconjugated anticancer drugs, including PG and PTX, as well as conjugated anticancer drugs (PG-LHRH and PTX-LHRH), were made at working concentrations of 0.5 mg/mL and then loaded into the device by incubating the drug solutions for 72 h in a refrigerator (in the dark, to avoid UV light-induced degradation).

##### Fabrication of Nonporous MNP-Modified PDMS Structure

To make PDMS substrates, a silicone elastomer base (Sylgard 184) was mixed with a silicone elastomer curing agent in a 10:1 volume ratio and thoroughly mixed. The mixture was degassed for an hour at −20 inHg in an Isotemp vacuum oven (model 280A, Fisher Scientific, Waltham, MA, USA). Magnetite nanoparticles (5 wt.%) were added to the mixture and degassed again. A three-dimensional solid mold was then filled with droplets of the resultant combinations. The samples were degassed for 15 min to eliminate any excess air bubbles created during the casting process. Subsequently, the samples were cured at 60 °C for 4 h and left to cool to room temperature (25 °C) before the nonporous MNP-modified PDMS structures were gently removed using surgical blades.

#### 2.2.3. Characterization of MNP-Modified PDMS

##### Fourier-Transform Infrared Spectroscopy (FTIR)

An FTIR spectrometer (Shimadzu in Kyoto, Japan) equipped with a horizontal attenuated total reflection (ATR) accessory was used to investigate the interaction between PDMS and magnetite nanoparticles. The sample was placed in the spectrometer, and an infrared beam was directed towards it. The interferogram collected by the spectrometer was Fourier transformed to generate the FTIR spectrum. This spectrum was then analyzed to identify the characteristic absorption bands and functional groups of PDMS and magnetite nanoparticles. The obtained spectra were in the range of 500 cm^−1^ to 4000 cm^−1^ and provided valuable information about the composition and structure of the materials, comparing neat PDMS and PDMS modified with magnetite nanoparticles (5% *w*/*w*).

##### Scanning Electron Microscopy (SEM)

A scanning electron microscope equipped with an Energy Dispersive Spectroscopy (EDS) detector for elemental analysis (JSM-7000F, Tokyo, Japan) was used to study the surface microstructure of the MNP-modified PDMS structure. The samples were prepared by fixing them onto SEM stubs using conductive adhesive. The samples were then sputter-coated with a thin film of gold using a sputter coater (EMS Q150R rotary pumped coater) to facilitate scanning electron microscopy (SEM) imaging. The coated samples were placed in the SEM chamber, and the electron beam was focused on their surfaces. The backscattered electrons emitted from the samples were detected and used to generate high-resolution images. The SEM images were analyzed to characterize the microstructure of the PDMS magnetite nanoparticles structures.

##### Thermogravimetric Analysis

Thermogravimetric Analysis (TGA) (NETZSCH-Geratebau GmbH in Germany) was used to assess the thermal stability and decomposition characteristics of the MNP-modified PDMS. Each sample weighing ~10 mg was heated in Al_2_O_3_ 85 µL in a crucible from an initial temperature of 25 °C to a final temperature of 800 °C at a heating rate of 20 ℃/min with 20 mL/min purified nitrogen purging. Data were analyzed and recorded using the Proteus analysis program (NETZSCH-Geratebau GmbH, Selb, Germany).

##### Differential Scanning Calorimetry 

Differential Scanning Calorimetry (DSC) (NETZSCH-Geratebau GmbH, Selb in Germany) was used to measure the heat emitted or absorbed by the materials. Exactly 10 mg of samples were placed into aluminum pans and sealed. The DSC analysis was conducted in a 25 to 400 °C temperature range with a continuous flow of nitrogen (20 mL/min). An empty hollow pan served as the reference. The exothermic peaks were analyzed using the Proteus thermal analysis program.

#### 2.2.4. In Vitro Drug Release Studies

In vitro drug release studies were conducted to analyze the release patterns of conjugated and unconjugated drugs from the MNP-modified microporous PDMS system. Each device loaded with 0.5 mg/mL of drugs (PG, PG+LHRH, PTX, and PTX+LHRH) was suspended in a centrifuge tube containing 1 mL of PBS buffer at pH 7.40 (which mimics the physiological conditions of the body), and the drug release studies were carried out at temperatures of 37, 41, and 44 °C for 30 days according to the method reported by Aina et al. [24].

#### 2.2.5. In Vitro Drug Release Kinetics 

The data obtained from the in vitro drug release studies was fitted with the Korsmeyer–Peppas model to determine the drug release kinetics. The Korsmeyer–Peppas model is a mathematical equation that describes the rate at which a polymeric material elutes its content. This model, also called the power law model, indicates a correlation between the time it takes for an active agent to release and its release rate. This is given by [25]:

(3) $$\frac{M_{t}}{M_{\infty }}=kt^{n}$$

In this equation, M_∞_, *M_t_*, *t*, *k*, and *n* represent the quantity of drug released at equilibrium, the amount released in time t, the duration of the drug release, the release rate constant, and the release exponent, respectively. The equation can alternatively be written in terms of concentration, with *C_t_* being the concentration of the active agent at time t and *C*_∞_ denoted as shown below.

(4) $$\frac{C_{t}}{C_{\infty }}=kt^{n}$$

Taking the natural logarithm of both sides of Equation (2),

(5) $$In\left(\frac{C_{t}}{C_{\infty }}\right)=Ink+nlnt$$

Hence, in this way, the power law exponent *n* was determined from the slope of plots of *ln*($C_{t}$/$C_{\infty })\;versus\;lnt.$

Once the *k* and *n* values are obtained, they can be used to interpret the drug release behavior. The release exponent n provides information about the release mechanism. For example, if *n* equals 0.5, it indicates Fickian diffusion-controlled release, while values greater than 0.5 suggest non-Fickian or anomalous transport.

#### 2.2.6. Cell Culture

The MDA-MB-231 cell line used in the research was acquired from the American Type Culture Collection (ATCC, Manassas, VA, USA) and cultured according to the supplier’s instructions.

#### 2.2.7. Cell Viability Studies

About 5 × 10^5^ MDA-MB-231 cells were seeded on coverslips (CELLTREAT, Pepperell, MA, USA) within 12-well culture plates and allowed to adhere overnight with drops of L15^+^ media (Leibovitz’s 15 medium with cell medium supplement of FBS and penicillin/streptomycin) gently added. Cells were left in the media to attach to the coverslips for 12 h.

Next, the implantable devices were introduced into the 6-well plates containing the seeded cells. The samples were incubated with the devices inside an incubator at 37 °C. The effect of drugs released from the system on cell viability was investigated via the Alamar Blue (AB) Assay throughout incubation durations of 0, 6, 24, 48, 72, 96 h (with the drugs).

Cell viability and cytotoxicity were assessed using the AB assay. This was achieved using the microplate reader. To perform the assay, the culture media were replenished with 1 mL of 10% AB solution. A 100 μL of the cell solution was incubated with AB solution (ABS) at 37 °C inside an opaque 96-well plate, and the microplate readings were obtained. The percentage of AB reduction and the percentage (%) of cell growth inhibition were determined.

#### 2.2.8. Flow Cytometry: Evaluation of Apoptosis Induction

Using flow cytometry, we identified the mechanism of cell death in MDA-MB-231 cells. In the experiment, 100 µL of MDA-MB-231 cells at a concentration of 3 × 10^5^ cells/mL were added to 6-well plates and incubated at 37 °C for 24 h. The cells were then treated with microporous membrane devices containing different drugs, namely prodigiosin, paclitaxel, LHRH-conjugated prodigiosin, and LHRH-conjugated paclitaxel of a working concentration of 0.5 mg/mL for another 24 h.

To measure the apoptosis rate, the cells were digested with trypsin (without EDTA), washed with PBS, and suspended in a binding buffer at 1~5 × 10^5^ /mL concentration of cells. A flow tube, capable of holding 5 mL of cell suspension, was filled with 100 µL of the cells. Subsequently, 5 µL of Annexin V EGFP was added to the flow tube, and the mixture was incubated in complete darkness for 5 min. Finally, 10 µL of propidium was added, followed by the addition of exactly 400 µL of PBS.

#### 2.2.9. Targeted Drug Delivery System for Triple-Negative Breast Cancer: Schematic Representation and Process Description

**Figure 1:**
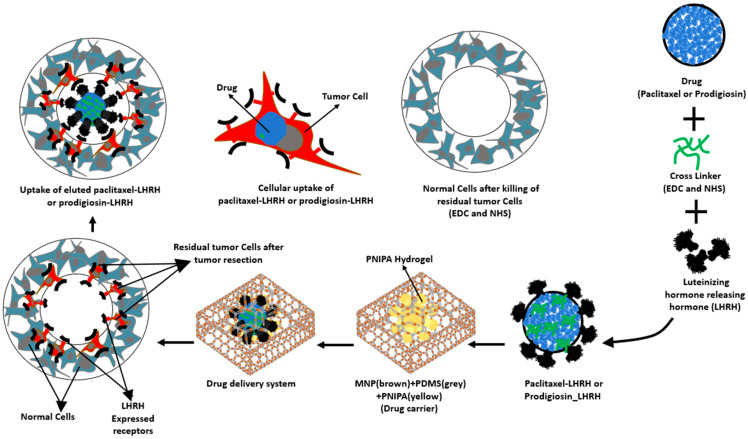
Schematic image describing the conjugation of prodigiosin or paclitaxel with LHRH, incorporation of the conjugated drug into the drug carrier, enhancement of uptake of the eluted conjugated prodigiosin or paclitaxel, and how the LHRH conjugation can enable the specific targeting of over-expressed LHRH receptors on any residual tumor tissues left after tumor resection.

Figure 1 shows a schematic representation of the process enabling the specific targeting of over-expressed LHRH receptors. First, conjugation of prodigiosin or Paclitaxel with LHRH involves chemically linking either prodigiosin or Paclitaxel to LHRH. The conjugated prodigiosin, or paclitaxel, is then incorporated into the magnetite nanoparticle-modified polydimethylsiloxane drug carrier and implanted after tumor resection. Next is the controlled release of the conjugated prodigiosin, or paclitaxel, for efficient uptake by triple-negative breast cancer cells expressing elevated levels of LHRH receptors on the cell membrane. This is particularly relevant for any residual tumor tissues that may remain after tumor resection.

In this system, Paclitaxel, a chemotherapy medication used to treat a variety of cancers, including ovarian, breast, lung, and pancreatic, and Prodigiosin, a natural red pigment produced by certain strains of bacteria such as Serratia marcescens with anticancer and immunomodulatory properties, both serve as the anticancer drugs. LHRH-mediated targeting is expected to increase the specificity and uptake of the drug, thereby improving treatment efficacy. Magnetite nanoparticles are integral to controlled drug release systems due to their ability to respond to external stimuli. PDMS serves as a versatile and protective biocompatible polymer matrix for encapsulating drugs.

#### 2.2.10. Data Analysis

The data obtained were expressed as the mean and standard deviation. A one-way analysis of variance (ANOVA) was done using the IBM statistical package for social science (SPSS) version 29, (IBM Corp, Armonk, 10504-1722, NY, USA) and was used to compare the groups. The *p* < 0.05 values were regarded as statistically significant.

## 3. Results

### 3.1. FTIR Analysis of MNP-Modified PDMS-Based Substrates

**Figure 2:**
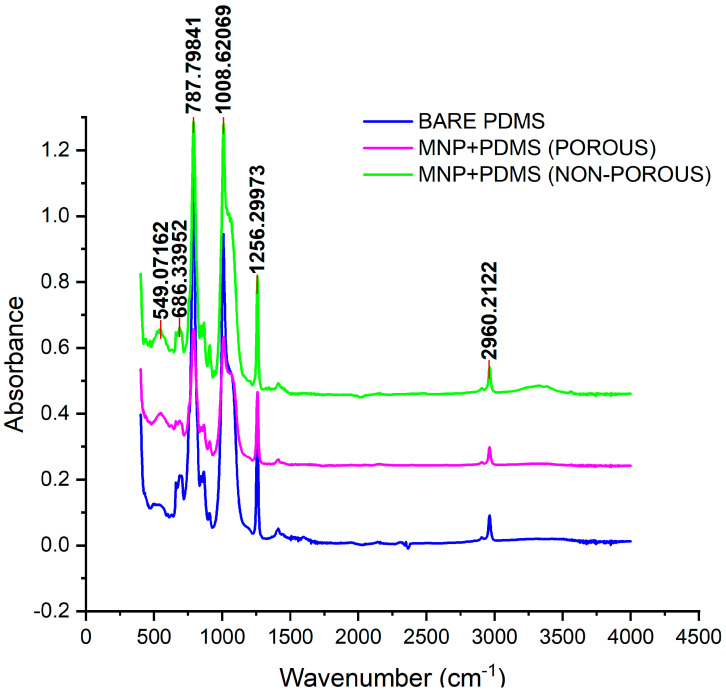
FTIR spectra of porous and nonporous MNP-modified PDMS-based substrates.

Fourier-transform infrared spectroscopy (FTIR) is a potent technique for identifying and describing the chemical characteristics of materials such as polymers like MNP-modified PDMS, as shown in Figure 2. FTIR was performed using Attenuated Total Reflectance Fourier-Transform Infrared Spectroscopy (ATR-FTIR) in the 4000–400 cm^−1^ wavenumber range. The two prominent peaks seen between 549.07 and 686.34 cm^−1^ are typically attributed to the Fe-O stretching vibration mode associated with the metal–oxygen interactions in the crystal structure of magnetite nanoparticles.

The IR peaks at 787.80 cm^−1^ for Si-C stretching and CH_3_ rocking in Si-CH3 bonds, 1008.62 cm^−1^ for Si-O-Si bond stretching vibrations, 1256.30 cm^−1^ for Si-CH_3_ bond stretching vibrations, and 2960.21 cm^−1^ for asymmetric CH_3_ stretching in Si-CH_3_ bonds correspond to the IR peaks of PDMS. All of these revealed the successful fabrication of MNP-modified PDMS, as the observed peaks corresponded to those of previous studies [26,27,28,29,30,31,32].

### 3.2. Scanning Electron Microscopy

**Figure 3:**
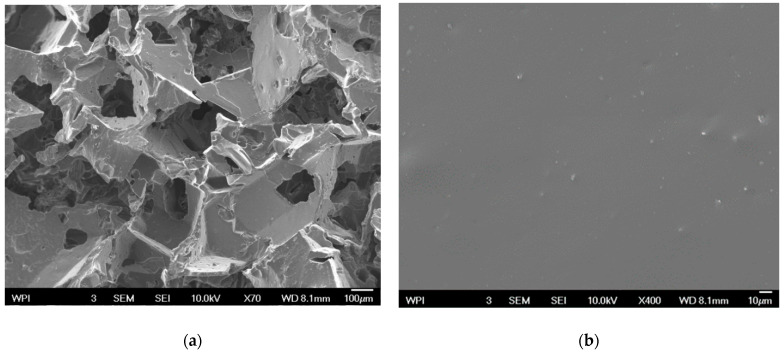
SEM images of MNP-modified PDMS-based surfaces at ×70 and ×400 magnifications; (**a**) porous and (**b**) nonporous.

The morphology and structure of the porous and nonporous MNP-modified PDMS scaffolds were described using SEM. Images from SEM are presented in Figure 3 to reveal the overall porosity levels. The MNP-modified PDMS had a porous structure with a dispersion of porosity throughout the MNP-modified PDMS matrix. In contrast, the nonporous MNP-modified PDMS structure had plain, smooth, and relatively featureless surfaces without significant irregularities. The magnetite nanoparticle clusters were uniformly distributed throughout the MNP-modified PDMS matrix.

### 3.3. Thermo-Gravimetric Analysis of MNP-Modified PDMS-Based Substrates

**Figure 4:**
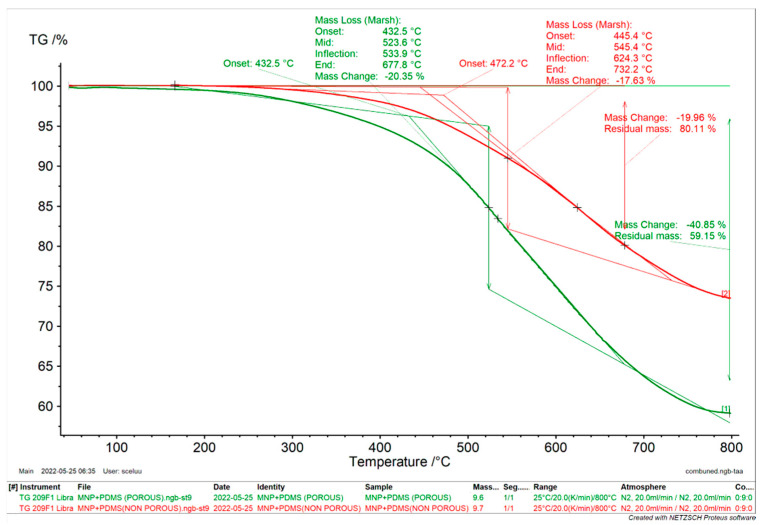
TGA curves of porous and nonporous MNP-modified PDMS-based substrates.

TGA is a valuable method for studying how materials behave during thermal degradation as a function of temperature. Figure 4 shows the results of a TGA analysis of the thermal stability of a porous and nonporous MNP-modified PDMS. While the nonporous MNP-modified PDMS had a mass change of 19.96%, the porous MNP-modified PDMS recorded a mass change of −40.85%. The nonporous MNP-modified PDMS may have had reduced mass loss due to the lack of pores that could enhance the decomposition process.

The residual mass of the porous MNP-modified PDMS was 59.15%, and that of the nonporous MNP-modified PDMS was 80.11%. The nonporous MNP-modified PDMS had a higher residual mass, which means that as it decomposes, it leaves behind more solid residue. Additionally, the porous and nonporous MNP-modified PDMS had onset temperatures of 432.5 and 472.2 °C, respectively. The greater decomposition onset temperature for nonporous MNP-modified PDMS suggests that it can resist higher temperatures before starting to decompose. These differences are essential when selecting PDMS materials for applications involving exposure to elevated temperatures. The results of this study corroborate with those of previous studies [29,30].

### 3.4. Differential Scanning Calorimetry of MNP-Modified PDMS-Based Substrates

**Figure 5:**
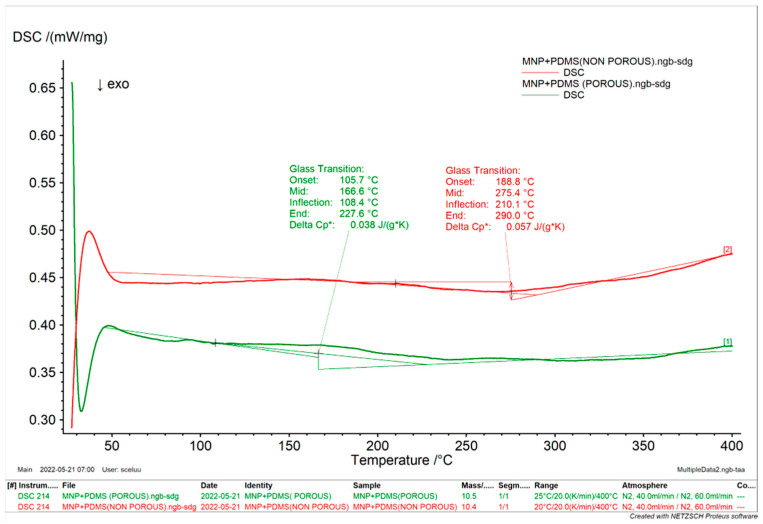
DSC thermogram of MNP-modified PDMS.

DSC gives details of the physical characteristics of the samples, such as whether they are crystalline or amorphous [33]. In our study, the DSC results (Figure 5) revealed significant distinctions between porous and nonporous MNP-modified PDMS. Porous MNP-modified PDMS exhibited an onset temperature of 105.7 °C. In comparison, nonporous MNP-modified PDMS had a higher onset temperature at 188.8 °C, signifying that the latter initiates thermal transitions at a slightly elevated temperature. Further examination of the inflection points demonstrated that porous MNP-modified PDMS reached 108.4 °C. In contrast, nonporous MNP-modified PDMS exhibited an inflection point at 210 °C, suggesting that the heat capacity change in nonporous MNP-modified PDMS occurs at a somewhat lower temperature, possibly due to differences in porosity.

Additionally, nonporous MNP-modified PDMS displayed a notably higher end temperature of 290.0 °C compared to 227.6 °C for porous MNP-modified PDMS, indicating its capacity to endure higher temperatures without significant thermal transitions. The change in heat capacity (ΔCP) further distinguished the two materials, with porous MNP-modified PDMS at 0.038 J/gK and nonporous MNP-modified PDMS at 0.057 J/gK, suggesting a more pronounced change in heat capacity during the thermal transition of nonporous MNP-modified PDMS.

### 3.5. Drug Release

**Figure 6:**
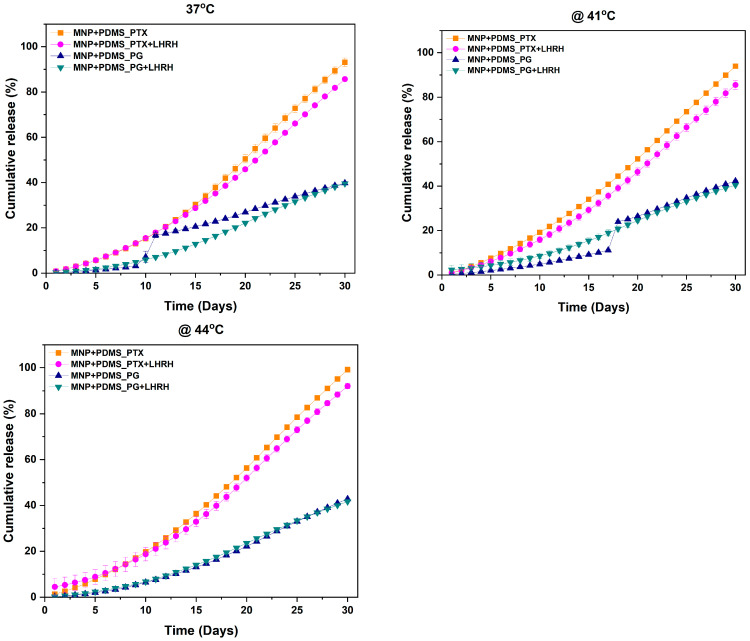
Cumulative drug release from magnetite nanoparticle-modified microporous poly-di-methyl-siloxane (PDMS) system in a buffer of pH 7.4 at 37, 41, and 44 °C.

Figure 6 shows the drug release profile of conjugated (PG+LHRH, PTX+LHRH) and unconjugated PG and PTX) drugs from MNP-modified microporous PDMS devices at 37, 41, and 44 °C. The findings indicated that temperature alterations significantly affected the device’s drug release performance. The release profile also showed that the greatest total drug release occurred at 44 °C, while the lowest drug release was observed at 37 °C, as illustrated in Figure 6.

The drug released from the device at temperatures of 37, 41, and 44 °C exhibited an initial fast release followed by a period of slower release. At 44 °C, approximately 99.24 and 92. 03% of the PTX and PTX+LHRH were released from the device after 30 days. Meanwhile, the cumulative drug release after 30 days at 41 was 93.94% for PTX and 85.48% for PTX+LHRH, and those at 37 °C were 93.10 and 85.66% for PTX and PTX+LHRH, respectively. The release of conjugated and unconjugated prodigiosin from the device was also observed under the same conditions. The release of PG and PG+LHRH were slower throughout the study period and at all the temperatures considered in this study, particularly 42.83 and 41.72 after 30 days at 44 °C, and approximately 42.31 and 40.46% were released at 41 °C after 30 days. However, the release of PG and PG+LHRH at 37 °C were 39.76 and 39.58, respectively.

### 3.6. In Vitro Drug Release Kinetics 

The data from the drug release studies were fitted to the Korsmeyer–Peppas equation to analyze the kinetics of drug release from drug-loaded MNP-modified porous PDMS devices at various temperatures. The release exponent (n) was then determined with a regression coefficient (R^2^).

**Figure 7:**
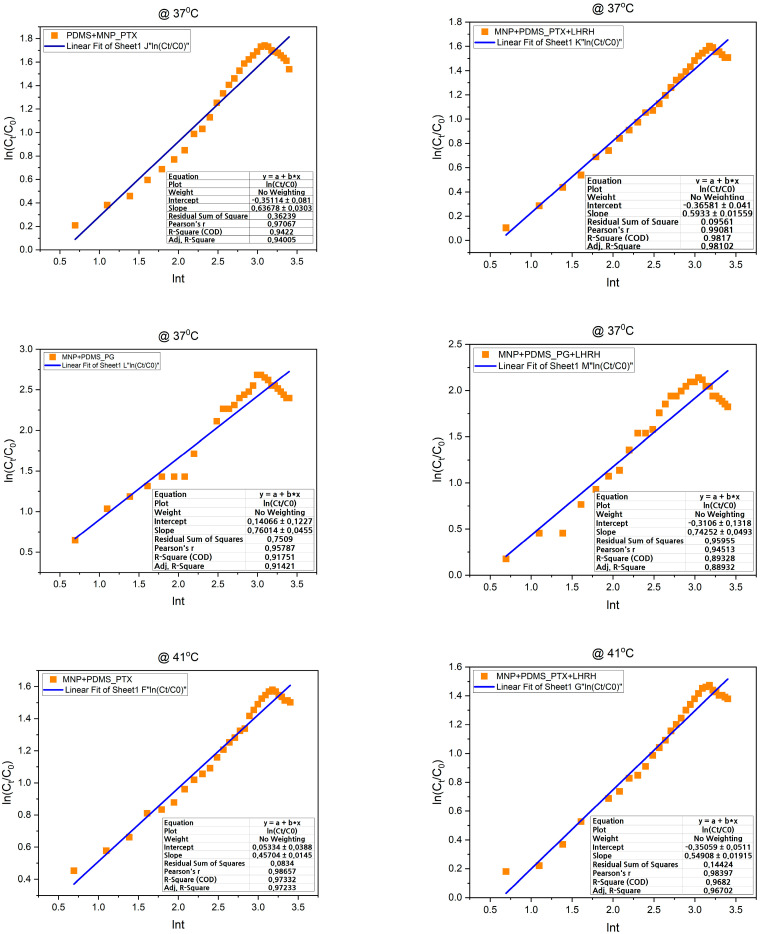

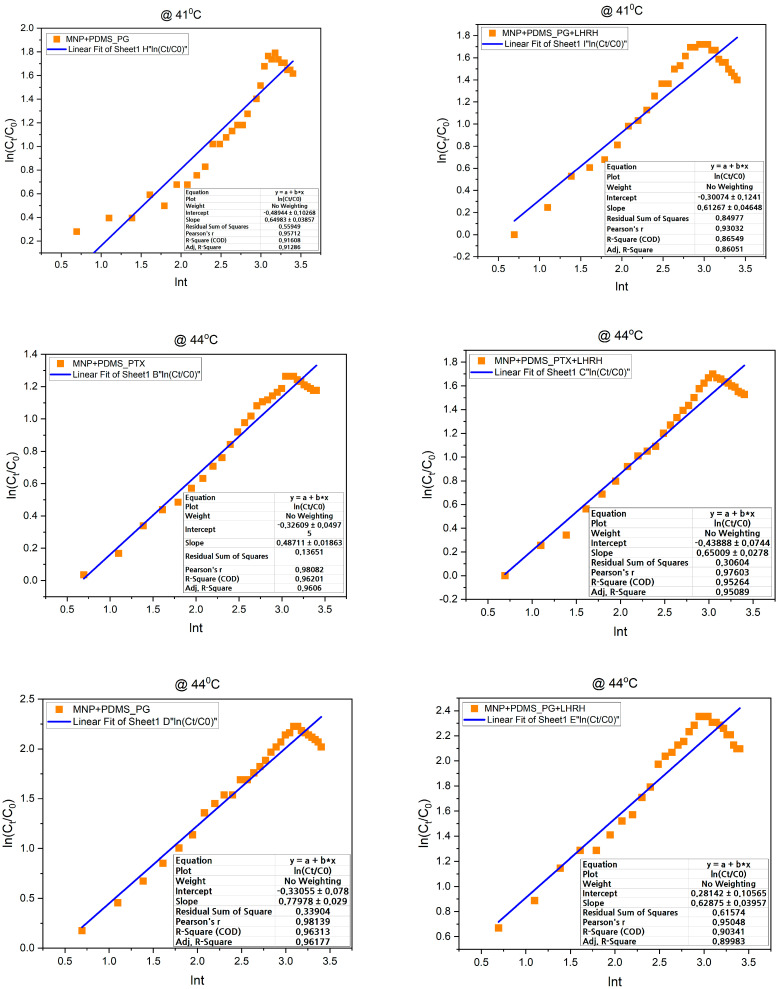
Korsmeyer and Peppas model drug release kinetics study showing anomalous (non-Fickian diffusion) drug release models at different temperatures (37, 41, and 44 °C) and for different samples used.

As shown in Figure 7, the Korsmeyer–Peppas model gave descriptive data on the release kinetics and mechanism of drug release. For MNP+PDMS_PTX, MNP+PDMS_PTX+LHRH, MNP+PDMS_PG, and MNP+PDMS_PG+LHRH at 37, 41, and 44 °C, the release exponents n > 0.45 and n < 0.89 revealed non-Fickian anomalous release.

### 3.7. Cell Viability and Cytotoxicity Assessment

**Figure 8:**
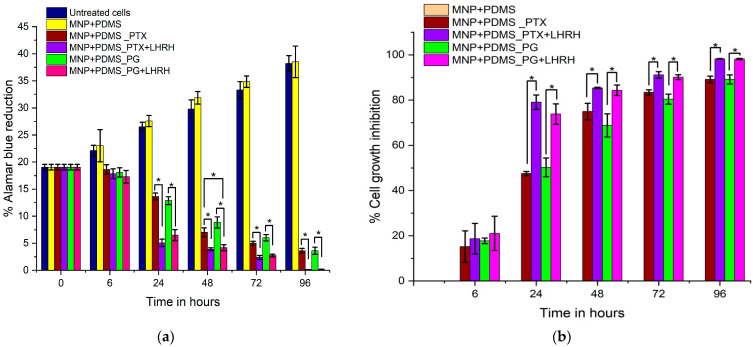
Assessment of MDA-MB-231 cell viability and cytotoxic effects of various drugs including PG, PTX, PG+LHRH, PTX, and PTX+LHRH administered via the MNP-modified microporous PDMS system at 6, 24, 48, 72, and 96 h after treatment. (**a**) % reduction in Alamar blue, (**b**) % cell growth inhibition. * Indicates that the mean difference is significant at *p* < 0.05.

Figure 8 presents the results of cell viability following the administration of drugs through the MNP-modified microporous PDMS system. The percentage of Alamar Blue reduction showed a progressive increase in both the untreated cell group and the MNP+PDM (without drug) group over the entire study period, as seen in Figure 8a. However, the drug-loaded groups significantly reduced the percentage of Alamar Blue throughout the treatment periods. On day 6, there were no differences in the percentage of Alamar Blue reduction (*p* > 0.05) between any of the groups. At 24, 48, 72, and 96 h, the groups loaded with conjugated drugs (PG+LHRH and PTX+LHRH) had a significantly higher percentage Alamar Blue reduction (*p* < 0.05) than the groups loaded with unconjugated drugs (PG and PTX). However, there were no significant differences (*p* > 0.05) among the unconjugated drug groups. Similar trends were observed for percentage cell growth inhibition (Figure 8b) for 24, 48, 72, and 96 h. Meanwhile, at 6 h, there were no differences in the percentage of cell growth inhibition (*p* > 0.05) between any of the groups. Additionally, throughout the study, the MNP+PDMS (without drug) group demonstrated a steady rise in the percentage of cell growth inhibition.

### 3.8. Effect of Treatments on the Induction of Apoptosis

**Figure 9:**
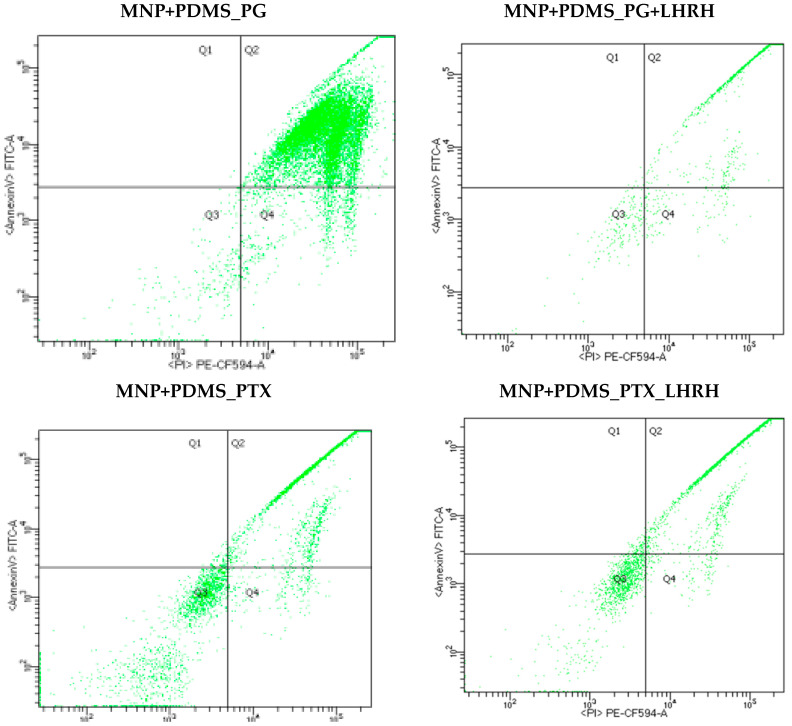
Flow cytometry analysis of apoptosis of MDA-MB-231 cells (3 × 10^5^ cells/mL) after culture for 3 days on Leibovitz’s medium supplemented with FBS. Horizontal Axis represents <AnnexinV> FITC-A while Vertical Axis represents <PI> PE-CF594-A. Apoptosis was assessed by the annexin V-FITC/PI double staining analysis following exposure to various treatments, including PG, PG+LHRH, PTX, and PTX+LHRH for 24 h. The upper right quadrants show late-stage apoptotic cells, characterized by their positive FITC-annexin V binding and uptake of propidium iodide. The lower right quadrants indicate early-stage apoptotic cells, which were positive for FITC-annexin V but negative for propidium iodide.

Flow cytometry assay was carried out to determine whether apoptosis or necrosis was the cause of the observed paclitaxel- and prodigiosin-induced cell growth inhibition effect. This study involved treating the cells with both conjugated (PG+LHRH and PTX+LHRH) and unconjugated (PG and PTX) drugs delivered through the device (system). Subsequently, the cells were stained with annexin V-FITC and propidium iodide for flow cytometry analysis. As shown in Figure 9, the flow cytometry results revealed distinct cell populations based on the presence or absence of annexin V-FITC or propidium iodide. In each panel, the lower left quadrant indicates viable cells, which are characterized by being negative for both FITC-annexin V and propidium iodide (PI). The lower left quadrant in each panel indicates viable cells, showing a negative status for both FITC-annexin V and propidium iodide (PI). The upper right quadrants display late-stage apoptotic cells, identified by their positive FITC-annexin V binding and uptake of propidium iodide. Lastly, the lower right quadrants represent early-stage apoptotic cells, which were positive for FITC-annexin V but negative for propidium iodide.

## 4. Discussion

Drug release is essential for treatment success and for achieving the highest level of patient compliance [34]. Figure 6 presents the drug release profiles of both conjugated (PG+LHRH, PTX+LHRH) and unconjugated (PG, PTX) drugs from MNP-modified microporous PDMS devices at varying temperatures (37, 41, and 44 °C). The term “drug release” describes the mechanism by which drug particles move from their initial location within the polymer system to the external surface of the polymer and subsequently into the surrounding release medium [35]. The cumulative drug release profile provides valuable insights into the performance of drug delivery systems. The findings of this study highlighted the significant influence of temperature alterations on the drug release performance of the device. Understanding the impact of temperature on drug release profiles is crucial in designing effective drug delivery systems. The release profile indicated that the highest total drug release was recorded at 44 °C, while the lowest was observed at 37 °C, as evidenced in Figure 6. This is because, at higher temperatures, the molecules in the system have greater kinetic energy, leading to increased molecular movement and diffusion. This finding is consistent with the results of earlier studies, which reported that raising the temperature facilitated the release rate [24,36,37,38,39]. This crucial finding demonstrates the importance of temperature in regulating drug release dynamics. It offers new possibilities for developing temperature-modulated drug delivery systems with special and regulated releases.

Moreover, evaluating drug release profiles at various temperatures (37, 41, and 44 °C), as depicted in Figure 6, demonstrated consistent trends across the studied drugs. A slower, sustained release followed an initial rapid release phase. This observation implies the possibility of an elevated drug concentration close to the polymer surface, which could aid in the first rapid release of the drugs.

In addition, the results revealed the significant impact of temperature on drug release profiles from the MNP-modified microporous PDMS devices, particularly evident in the release of PTX (paclitaxel) and PTX+LHRH (paclitaxel with luteinizing hormone-releasing hormone) (Figure 6). At elevated temperatures, notably 44 °C, a significant cumulative drug release, approximately 99.24% for PTX and 92.03% for PTX+LHRH after 30 days, was observed. Likewise, at 41 °C, the cumulative release for PTX was 93.94%, whereas PTX+LHRH exhibited an 85.48% release. At 37 °C, lower cumulative drug releases were noted, with PTX at 93.10% and PTX+LHRH at 85.66%. In contrast, the release profiles of conjugated and unconjugated prodigiosin (PG and PG+LHRH) from the same devices were significantly slower across all temperature conditions. Specifically, after 30 days at 44 °C, approximately 42.83% of PG and 41.72% of PG+LHRH was released. Similarly, at 41 °C, the release percentages were approximately 42.32% for PG and 40.46% for PG+LHRH. At 37 °C, the cumulative release of PG and PG+LHRH dropped significantly to 39.76% and 39.58%, respectively. The above differences in the release profiles of the prodigiosin and paclitaxel groups could be due to differences in the molecular weights of the drugs. The molecular weight of paclitaxel is 853.9 g/mol, while that of prodigiosin is 419.9 g/mol [40]. This difference could also be due to various factors, including the physicochemical properties of the drugs, their interactions with the MNP-modified PDMS material, and their solubility. Despite these distinctions, the results consistently exhibited sustained release, irrespective of the temperature. This sustained drug delivery observed throughout the study suggests that this system has the potential to maintain a reliable delivery profile and prevent drug leakage during the delivery process. Moreover, the conjugated drug had a better drug release profile compared to the unconjugated drugs. This suggests that drug conjugation can impact drug release profiles. Drug conjugation is often used to modify drug properties, including solubility, stability, and release kinetics [41,42]. Also, conjugation might alter the drug’s interaction with the MNP-modified PDMS or affect the diffusion rate, leading to increased drug release.

Next, the study utilized the Korsmeyer–Peppas model, a well-established tool for characterizing drug release from polymeric structures [43,44]. The kinetics of drug release were determined by applying this model to the drug release data obtained from drug-loaded MNP-modified porous PDMS devices across varying temperatures. Specifically, the release exponent (n) and regression coefficient (R^2^) derived from the Korsmeyer–Peppas equation provided important data about the release mechanism. Across MNP+PDMS_PTX, MNP+PDMS_PTX+LHRH, MNP+PDMS_PG, and MNP+PDMS_PG+LHRH at temperatures of 37, 41, and 44 °C, the calculated release exponents (n) fell within the range of >0.45 and <0.89 (Figure 7). This range indicates a non-Fickian anomalous release mechanism, suggesting that drug release was influenced by a combination of diffusion and erosion [29,33]. The release kinetics can also be affected by other processes, such as swelling of the polymer matrix and drug-polymer interactions. Moreover, it was shown that the diffusion coefficients and interactions between the drug and the MNP-modified PDMS matrix were unaffected by temperature variation (37, 41, and 44 °C), allowing for consistent release behavior. The above results have implications for drug delivery system design, temperature control strategies, and the potential for modifying drug release profiles. Thus, the results of this study will be helpful in guiding the design of more robust drug delivery systems that can meet specific therapeutic requirements, such as sustained release over an extended period or controlled release with temperature-dependent responses.

In addition, the impact of drug delivery via the MNP-modified microporous PDMS system on cell viability, as measured by the percentage of Alamar Blue reduction and cell growth inhibition, is shown in Figure 8. Organic materials are commonly coated with MNPs, including polymers and fatty acids, to improve their stability and biocompatibility for therapeutic applications [45]. For the successful treatment of human diseases, it is imperative to establish an appropriate therapeutic index that not only signifies the drugs’ specific effects on target cells but also ensures the absence of clinically significant impacts on the host [46]. In this study, we evaluated the efficacy of two conjugated drugs (PTX+LHRH and PG+LHRH) and their single forms (PG and PTX) when delivered through this unique drug delivery system. By examining the decrease in Alamar Blue and the inhibition of cell growth, we were able to ascertain their efficacy. The Alamar Blue assays are essential in pharmacology and cell biology research to assess cytotoxicity or the ability of a drug to prevent cell growth. A natural trend in cellular metabolic activity and viability is suggested by the steady increase in the percentage of Alamar Blue that was seen at 6, 24, 48, 72, and 96 h in the MNP+PDM (without drugs) group and the untreated cell group (Figure 8a). However, low percentage Alamar Blue reduction levels observed in the drug-loaded groups at 24, 48, 72, and 96 h signal that fewer metabolically active and viable cells were present, which indicates that the drugs significantly suppressed cell growth. The efficacy of the conjugated and unconjugated drugs observed in this study is due to the carrier system, which provided sustained release and increased the therapeutic index throughout the study. These results suggest that the drug delivery system had a time-dependent impact on the treatment outcome, leading to enhanced metabolic activity as time progressed. The anticancer activity of sustained release has been highlighted in several studies [9,47,48,49,50].

Furthermore, the results showed that PG+LHRH and PTX+LHRH presented greater inhibition of MDA-MB-231 cells compared to PG and PTX (Figure 8b). The significant differences in percentage cell growth inhibition and Alamar blue reduction between the conjugated and unconjugated drugs at various time points (24, 48, 72, and 96 h) suggest that the conjugation of the drugs with LHRH significantly enhanced the activity of the drugs. With expression in various malignant cells, such as endometrial, ovarian, and triple-negative breast cancer (TNBC) cells, LHRH has been recognized for its role in the negative autocrine/paracrine regulatory mechanism of cell proliferation [20,21,22]. This targeting moiety has high specificity for receptors found in TNBC cells, resulting in a tailored and effective drug delivery system. The fundamental concept of drug targeting involves transporting a substantial amount of medication to a specific location while reducing its presence in areas not intended for treatment. This approach is instrumental in enhancing the therapeutic impact of the drug while mitigating the adverse effects associated with interactions at multiple targets, elevated doses, and unintended concentrations in nontargeted regions [51]. The findings of this study align with a prior report, which showed that the enhanced binding of LHRH-conjugated drugs to TNBC cells and tissues in both in vitro and in vivo studies improve specific targeting and efficacy [20,21,22]. The presence of identifiable molecules on the surface of a drug enables its specificity. This is crucial because the drugs need to penetrate the nucleus, mitochondria, endoplasmic reticulum, and various organelles [52].

Moreover, the flow cytometry results, as shown in Figure 9, suggested that the cell death observed in this study was primarily due to early- and late-stage apoptosis. Early-stage apoptosis is marked by changes in the cell membrane, such as phosphatidylserine translocation, while late-stage apoptosis is characterized by increased cell membrane permeability.

Apoptosis is a is a programmed cell death mechanism that takes place in multicellular organisms under physiologically normal circumstances to support several biological systems, such as the immune system, tissue homeostasis, and cell turnover [53]. It is the most significant indicator of cancer treatment for various cancer cells. The ability of the treatment method to trigger apoptosis, either by targeting the excessively expressed antiapoptotic proteins or by inducing the release of proapoptotic molecules, is a key component of any therapeutic approach. It has been shown that paclitaxel induces apoptosis through several mechanisms.

Paclitaxel triggers apoptosis in different cell types through various signaling pathways, including the PI3K/RAC-serine/threonine-protein kinase (AKT), the epidermal growth factor receptor, and the mitogen-activated protein kinase (MAPK) pathway [54]. Paclitaxel has been found to potentially induce apoptosis in cancer cells through the activation of various pathways, including BRCA1/JNK and p38, as well as the activation of tumor suppressor genes like PTEN and p53 [55]. Also, taxanes inhibit the development of the cell cycle by impairing the centrosomal process, causing aberrant spindles, and suppressing spindle microtubule dynamics [56]. 

In addition, it has been reported that the mitochondria-cytochrome c pathway is primarily responsible for the prodigiosin-induced apoptotic response. When cytochrome c enters the cytoplasm, it activates procaspase-9, which can then activate other active caspases like caspase-3 and cause apoptosis [57].

## 5. Conclusions

In this study, we developed a modified poly-di-methyl-siloxane (PDMS) system for the delivery of conjugated drugs (PG+LHRH and PTX+LHRH) for the targeted suppression of triple-negative breast cancer cells. Our in vitro drug release kinetics studies demonstrated sustained and controlled extended-release profiles of the LHRH-conjugated drugs from the magnetite nanoparticle-modified microporous PDMS system. In addition, treatment with the drug-loaded MNP-modified microporous PDMS system substantially reduced the viability and proliferation of triple-negative breast cancer cells, indicating that our device holds great promise for therapeutic applications in TNBC treatment. The next stage of this research involves animal studies. The magnetite nanoparticle-modified poly-di-methyl-siloxane will be used in the form of implants after tumor resection. The luteinizing hormone-releasing hormone-conjugated drugs will be delivered directly to the target site through a sustained release mechanism from the implant. This is particularly relevant for any residual tumor tissues that may remain after tumor resection to prevent tumor recurrence. Due to its relatively large size, as observed in the SEM images, the carrier is not intended to be phagocytosed by individual cells. Rather, after the treatment is completed, the implant device will be surgically removed. This approach ensures that the drug delivery system effectively administers the treatment while in place and then is safely removed to avoid any long-term foreign body reactions.

In future studies, a more comprehensive material characterization is recommended.

## Data Availability

The data that support the findings of this study are available from the corresponding author upon reasonable request.
